# Gender Representations, Empathy, and Gender-Based Violence Awareness Among Medical Students: A Proposal for a Specific Training Program

**DOI:** 10.7759/cureus.65266

**Published:** 2024-07-24

**Authors:** Giulia Savarese, Luna Carpinelli, Giovanna Stornaiuolo, Stefano Bifulco, Giorgia Bruno, Marco Navarra

**Affiliations:** 1 Medicine and Surgery, University of Salerno, Baronissi, ITA; 2 Social Studies, University of Salerno, Baronissi, ITA

**Keywords:** gender-based violence, skill training, clinical empathy, college student, gender bias

## Abstract

Introduction: In recent decades, the topic of gender differences has become central to many areas of study, including medicine. The present study explored gender differences in empathy, gender role ideologies, and gender sensitivity among medical students, highlighting significant variations that can inform medical education and training programs.

Materials and methods: The study involved 155 students (52.1% male; mean age: 22.68±2.48 years) from the Department of Medicine, Surgery, and Dentistry of the University of Salerno in Baronissi, Southern Italy. Participants completed two standardized scales: the Jefferson Scale of Empathy (JSE) to assess empathy, and the Nijmegen Gender Awareness in Medicine Scale (N-GAMS) to evaluate gender awareness. Six open-ended questions were also included in the gender-based violence representations.

Results: The ANOVA analysis reveals significant differences in scores between male and female students across the N-GAMS scales, indicating a strong role of gender in these variations. These findings suggest the necessity for further research to understand the contributing factors and inform targeted interventions in medical education. Additionally, there are significant differences in compassionate care (Factor 2) and walking in the patient’s shoes (Factor 3), highlighting the substantial impact of gender on these latter aspects of empathy.

Conclusions: These gender differences have significant implications for medical education. Training programs should be tailored to address the specific needs and characteristics of both male and female students. For example, encouraging male students to maintain their gender sensitivity while challenging traditional gender role ideologies can promote a more inclusive approach to patient care. For female students, fostering confidence in their compassionate care abilities and providing opportunities to express empathy in diverse ways can help overcome societal constraints.

## Introduction

In recent decades, the topic of gender differences has become central to many areas of study, including medicine [1]. The relationship between doctor and patient is inherently based on trust and understanding. When a patient feels that the doctor truly understands his or her specific needs, he or she feels more secure and supported. This trust translates into better adherence to treatment, more open communication, and greater involvement in one's care pathway [2]. Furthermore, a greater knowledge of gender differences can help doctors overcome unconscious biases that may influence their clinical judgment. With proper training, doctors can recognize and correct these biases, ensuring that all patients receive the same attention and care, regardless of gender. It means seeing each patient as a unique individual with his or her own specific needs and characteristics. It is a step towards more personalized, inclusive, and fair medicine.

Empathy, defined as the ability to understand and share the feelings of others, and gender sensitivity, i.e., the ability to recognize and respond appropriately to the different health needs of men and women, are crucial qualities for health professionals [3]. Understanding how these qualities vary between medical students of different genders can offer important insights into improving medical education and, consequently, the quality of care provided.

Numerous studies [3, 4] have indicated significant gender differences in empathy levels among medical students. Women tend to report higher scores than men on measures of empathy. This phenomenon can be attributed to various factors, including social and cultural differences in education, gender expectations, and possible neurobiological variations [5,6]. Research indicates that societal and cultural norms play a significant role in shaping empathetic behaviors [6]. Women are often socialized to be more attuned to the emotions and needs of others, which can enhance their empathetic abilities. Studies [7,8] show that girls are encouraged to be nurturing and expressive about their feelings from a young age, while boys might be steered towards independence and stoicism. This social conditioning can lead to women developing stronger empathy skills as they grow up. On a biological level, neuroimaging studies [8] have identified structural and functional differences in male and female brains that may underlie variations in empathy. For example, women tend to have a higher density of mirror neurons, which are crucial for understanding and mirroring the emotions of others. Additionally, hormonal differences, such as higher levels of oxytocin in women, are linked to nurturing behaviors and empathy [8].

Gender expectations also play a crucial role [9]. Societal expectations that women should be more caring and emotionally responsive can lead to self-fulfilling prophecies, where women adopt and exhibit more empathetic behaviors to align with these expectations. Conversely, men might suppress empathetic responses to conform to traditional notions of masculinity.

These differences may influence how students relate to patients and handle clinical interactions. The way medical students perceive and understand gender is crucial to their ability to recognize and respond to gender-based violence. Studies have shown that medical students can exhibit gender biases and stereotypes that can negatively impact their interactions with patients and their understanding of gender-based issues [10,11]. For example, research has found that male medical students tend to hold more traditional views on gender roles and are less empathetic towards female patients, which can influence their approach to addressing gender-based violence [11,12]. 

How gender-based violence is represented in medical education can shape students' understanding, attitudes, and behaviors toward survivors [12,13]. It is crucial to examine whether medical curricula adequately address the complexities, prevalence, and impact of gender-based violence [12]. Additionally, exploring the portrayal of survivors and perpetrators in educational materials is essential to ensuring that harmful stereotypes or biases are not perpetuated [13,14].

Therefore, the main aim of this study was to explore gender representations and bias, empathy level, and representations of gender-based violence in medical students. Additionally, it intends to provide training on these issues for medical students.

By critically examining the representations of gender-based violence in medical education, we can identify areas for improvement and develop strategies to enhance education and training [15]. This may involve incorporating comprehensive and evidence-based content on gender-based violence, integrating survivor-centered approaches, and promoting empathy and cultural sensitivity among future healthcare professionals [1].

By shedding light on this issue, we can contribute to the ongoing efforts to improve medical education and equip future physicians with the necessary skills and knowledge to effectively address gender-based violence [10, 11]. Regarding curriculum content, it is crucial to include comprehensive, evidence-based training on gender-based violence, covering the different forms of abuse and the consequences for victims [10]. In addition, it is important to regularly update content to reflect the most recent developments in understanding gender-based violence [12].

When it comes to teaching methods, it is advisable to use interactive and participatory approaches that engage students in hands-on activities and guided discussions [16]. The inclusion of direct testimonies from survivors of gender-based violence can be particularly effective in fostering student empathy [17].

The impact on students' education is significant, as the representation of gender-based violence can influence their attitudes and behaviors [17,18]. Students need to develop a critical awareness of the challenges associated with gender-based violence and acquire skills to provide adequate support to victims [18,19].

Finally, interprofessional collaboration is key to addressing gender-based violence effectively [19,20]. Students should be exposed to interprofessional learning experiences that reflect the reality of managing gender-based violence in clinical practice [20].

## Materials and methods

Procedures

The survey participants were undergraduate students of the Department of Medicine, Surgery, and Dentistry, Scuola Medica Salernitana, of the University of Salerno in Baronissi, Southern Italy. Three hundred and twelve students participated in integrative teaching activities focused on gender medicine. At the end of these activities, the link to the Google Form (Google Inc., Mountain View, CA) containing the survey was sent by email. The completion of the survey was estimated at approximately 10 minutes. One hundred and fifty-five students (52.1% male; mean age: 22.68±2.48 years) responded to the survey. 

The survey had three distinct sections. The first section contained information on the purpose of the study, context, and informed consent. The data were anonymized and used to analyze the study in aggregate form. The second, quantitative section included two standardized scales, described in detail below.

The Jefferson Scale of Empathy (JSE)

The JSE [21] is a widely used tool in medical education and research to measure empathy among healthcare professionals, particularly medical students and physicians. The JSE consists of a 20-item self-reporting questionnaire that assesses an individual's perspective-taking ability, compassionate care, and understanding and communication with patients. It is designed to measure both cognitive and affective components of empathy, and respondents rate their level of agreement or disagreement with each statement using a seven-point Likert scale, with one (strongly disagree) and seven (strongly agree) for positively responded items and one (strongly agree) and seven (strongly disagree) otherwise. Therefore, the scores ranged from 20 to 140. Higher scores indicate greater empathy. It has been categorized into three subscales, including perspective-taking (10 positively responded items), compassionate care (eight negatively responded items), and walking in the patient's shoes (two negatively responded items).

Nijmegen Gender Awareness in Medicine Scale (N-GAMS)

The N-GAMS [22] is a psychometric tool developed to assess the level of gender awareness among medical professionals. The N-GAMS consists of a questionnaire that measures various dimensions of gender awareness, including knowledge, attitudes, and skills related to gender-sensitive healthcare. This scale is divided into three subscales: (1) gender sensitivity (GS): the degree to which healthcare professionals/medical students are sensitive and sympathetic to the impact of gender in medical practice (14 items); (2) gender-role ideology towards patients (GRIP): health care providers' stereotypical views towards male and female patients (11 items); and (3) gender-role ideology towards doctors (GRID): healthcare providers' stereotypical views towards male and female doctors (seven items). Answers are assessed on a five-point Likert scale ranging from one ("totally disagree") to five ("totally agree").

Open Questions on Gender-Based Violence

The third section for qualitative data collection comprised six open questions on gender-based violence which were as follows: 1. What types of violence do you think are most prevalent? 2. How do you imagine a violent person? 3. How would you react if you were the victim of violence? 4. What do you think are the causes of violence? 5. What are, in your opinion, the protective factors of violence? 6. In your opinion, how can violence be reduced?

Data analysis

IBM SPSS Statistics software for Windows, version 22.0 (IBM Corp., Armonk, NY) was used for the quantitative analysis. Descriptive analyses were conducted to examine the variables under study: participants' age, gender, JSE, and N-GAMS standardized scores. The answers to the open-ended questions were analyzed with a qualitative approach using T-Lab Plus software (T-LAB di Lancia Franco, Roccasecca, Italy) [23] to provide an in-depth understanding of the textual data, helping to uncover hidden themes and relationships between concepts. In particular, identifying text segments containing similar terms through co-occurrence and cluster analysis identifies the main themes emerging from the data [24].

## Results

Results of N-GAMS

Table 1 shows the results of the N-GAMS for the gender variable. The collected data show interesting gender differences in the mean scores for the three scales: GS, GRIP, and GRID. Male students tended to have higher average scores than female students. However, male scores also showed greater variability compared to female scores. These differences may indicate various underlying dynamics, including cultural, educational, and individual factors.

**Table 1:** Nijmegen Gender Awareness in Medicine Scale (N-GAMS) results for the gender variable GS: gender sensitivity; GRIP: gender role ideology toward patients; GRID: gender role ideology toward doctors

Sex		GS score	GRIP score	GRID score
Female	M	2.62	1.57	1.57
	SD	0.55	0.72	0.72
Male	M	2.95	2.26	2.20
	SD	0.83	1.18	1.27
Total	M	2.77	1.89	1.87
	SD	0.71	1.02	1.06

Gender Sensitivity

This score measures awareness and understanding of gender issues. Male students having higher scores could indicate greater awareness of gender issues, possibly due to a personal interest or specific training received. However, it is also possible that the differences in scores reflect variations in how males and females interpret and respond to the questions, with male students possibly feeling more confident in demonstrating high gender sensitivity.

Gender Role Ideology Toward Patients

This score reflects beliefs about appropriate gender roles regarding patients. Higher scores among male students could indicate a tendency to maintain or promote more traditional or specific gender roles. It may also suggest a different perception of the needs and expectations of patients based on gender, influenced by cultural or social factors.

Gender Role Ideology Toward Doctors

This score concerns beliefs about appropriate gender roles for doctors themselves. Higher scores among male students might reflect a more traditional view of gender roles within the medical profession, perhaps supporting a more patriarchal or hierarchical view. This could be influenced by personal experiences, role models, or cultural and social norms.

The ANOVA Analysis for N-GAMS

Table 2 presents the results of the ANOVA analysis, which tests for differences in scores between male and female students on the three different N-GAMS. The significant F-statistics and the very low p-values for each measure provide strong evidence that gender plays a role in the differences observed in these scores. These findings highlight the need for further investigation into the factors contributing to these differences and could inform targeted interventions in medical education to address any underlying causes.

**Table 2:** The ANOVA test for the Nijmegen Gender Awareness in Medicine Scale (N-GAMS) GS: gender sensitivity; GRIP: gender role ideology toward patients; GRID: gender role ideology toward doctors

			Sum of squares	Degree of freedom	Root mean	F	Sign.
GS score*Sex	Between groups	(Combined)	4.203	1	4.203	8.647	.004
	Within groups		72.901	150	.486		
	Total		77.104	151			
GRIP score*Sex	Between groups	(Combined)	18.354	1	18.354	19.604	<0.001
	Within groups		143.241	153	.936		
	Total		161.595	154			
GRID score*Sex	Between groups	(Combined)	15.030	1	15.030	14.480	<0.001
	Within groups		156.733	151	1.038		
	Total		171.763	152			

Results of JSE

The results from the JSE (Table 3) indicate that while perspective-taking (Factor 1) scores are nearly identical for both male and female students, there are more pronounced differences in compassionate care (Factor 2) and walking in the patient’s shoes (Factor 3). Female students generally exhibited lower averages with less variability in compassionate care and walking in patients’s shoes compared to their male counterparts. These findings suggest that gender differences are more evident in certain aspects of empathy, with male students displaying greater variability and higher average scores in specific factors. 

**Table 3:** The Jefferson Scale of Empathy (JSE) scale results for the gender variable M: average; SD: standard deviation

Sex		Perspective-taking (Factor 1)	Compassionate care (Factor 2)	Walking in the patient’s shoes (Factor 3)
Female	Mean	42.54	31.08	5.22
	SD	6.55	5.14	2.38
Male	Mean	42.58	33.40	6.78
	SD	8.84	7.57	2.86
Total	Mean	42.56	32.16	5.94
	SD	7.68	6.47	2.72

Perspective-Taking (Factor 1)

The nearly identical scores between male and female students suggest that both genders are equally capable of understanding others' perspectives. This factor might be less influenced by gender-specific traits and more by general cognitive and emotional development, which could be similarly emphasized in their education and socialization.

Compassionate Care (Factor 2)

Female students showing lower averages could indicate that they are more modest in self-assessing their compassionate behavior, possibly due to societal expectations of humility or self-criticism. The lower variability among female students might suggest a more consistent standard or approach to compassionate care, possibly influenced by gender norms that emphasize consistent caregiving roles for women.

Walking in the Patients’ Shoes (Factor 3)

Similar to Factor 2, this could reflect a more cautious self-assessment among female students or different experiences and interactions with patients that shape their empathy. Consistent lower variability might again point to a more uniform way female students experience and express empathy, possibly due to socialization that encourages a specific empathetic demeanor.

The ANOVA Analysis for the JSE Scale

The ANOVA results (Table 4) demonstrate that while there is no significant gender difference in perspective-taking (Factor 1), there are statistically significant differences in compassionate care (Factor 2) and walking in the patient’s shoes (Factor 3) between males and females. This implies that gender substantially impacts the scores for compassionate care and walking in the patient’s shoes but not for perspective-taking.

**Table 4:** The ANOVA test for the Jefferson Scale of Empathy (JSE) scale EMP: empathy

			Sum of squares	Degree of freedom	Root mean	F	Sign.
Factor1_ EMP*Sex	Between groups	(Combined)	.065	1	.065	.001	.974
	Within groups			153	59.373		
	Total			154			
Factor2_ EMP*Sex	Between groups	(Combined)	207.239	1	207.239	5.078	.026
	Within groups			153	40.809		
	Total			154			
Factor3_ EMP*Sex	Between groups	(Combined)	93.937	1	93.937	13.681	<0.001
	Within groups			153	6.866		
	Total			154			

Qualitative analyses

Table 5 presents an occurrence analysis of various words or concepts that emerged in the open-ended questions. The most frequently occurring words/concepts were "gender" (49 occurrences), "violence" (48 occurrences), and "psychological" (36 occurrences). This suggests the respondents often discussed different forms of violence, especially those with psychological or gender-based aspects. Other commonly appearing words related to types of violence, such as "physical" (31 occurrences), "verbal" (28 occurrences), and "sexual" (21 occurrences). This indicates the respondents considered various manifestations of violence.

**Table 5:** Occurrences (OCC) analysis of responses to six open questions

Item (n= 23)	OCC
Gender	49
Violence	48
Psychological	36
Person	32
Physics	31
Verbal	28
Aggressive	26
Sexual	21
Ignorant	10
Irascible	8
Gives	8
Violent	7
Maid	6
Manipulative	4
Narcissistic	4
Impulsive	4
Emotions	4
Easily	4
Female	4
Bullying	4
Bad	4
Physically	4
Frustrated	4

Respondents also frequently used words describing perpetrator characteristics, such as "aggressive" (26 occurrences), "ignorant" (10 occurrences), "irascible" (eight occurrences), "violent" (seven occurrences), and "manipulative" (four occurrences). This implies a focus on understanding the psychological profiles of those who commit violence.

Some words suggest the respondents discussed the impact of violence, such as "person" (32 occurrences), "women" (eight occurrences), "emotions" (four occurrences), and "frustrated" (four occurrences). This could indicate an interest in the experiences and perspectives of those affected by violence. A few words, like "domestic" (six occurrences) and "bullying" (four occurrences), point to specific contexts or settings where violence may occur.

We observed several interesting comments in the open responses: Question #1 regarding the most common types of violence indicated that physical and sexual violence, along with psychological and verbal violence, were considered the most common types of violence. For Question #2, which asked how you imagine a violent person, the answers were distributed among different clusters, indicating different perceptions of a violent person. However, many participants believed that an abusive person can be aggressive, manipulative, lacking empathy, and prone to anger and frustration. Question #3, which pertained to the reaction of a victim of violence, yielded several clusters of answers. However, many participants said that they would consider the option of reporting violence, seeking communication and support from those they are close to, and taking personal actions to seek help and protection. In Question #4, which asked about the causes of violence, the answers highlighted a variety of perceptions about the causes of violence. However, many participants pointed to ignorance, insecurity, past trauma, family problems, and social and cultural context as possible causes of violence. Question #5, which was regarding protective factors against violence, had limited answers, but they emphasized the importance of effective education, awareness-raising in schools and universities, education, and empathy as possible protective factors. For Question #6, which asked how to reduce violence, the answers are also limited, but they highlighted the importance of education, awareness-raising, and self-protection education as possible avenues.

## Discussion

This study investigated the differences between male and female medical students in terms of empathy, gender role ideologies, and gender sensitivity, revealing notable variations that can guide improvements in medical education and training. These results are consistent with various studies [6-11] showing that gender-specific socialization and cultural expectations significantly influence levels of empathy and gender sensitivity.

The analysis of the data obtained from the N-GAMS Scale and JSE Scale provides an interesting perspective on the mean differences between the sexes and the relationships between the variables. 

The results demonstrate no significant gender difference in perspective-taking (Factor 1), as male and female students scored nearly identically. This suggests that both genders possess a similar capacity for understanding others' perspectives, likely due to the general cognitive and emotional development emphasized in their education and socialization, which aligns with research indicating that perspective-taking is a fundamental component of empathy that may not be heavily influenced by gender-specific traits [7-9]. This suggests that medical education effectively fosters this aspect of empathy equally among male and female students.

Conversely, significant gender differences were observed in compassionate care (Factor 2) and walking in the patient’s shoes (Factor 3). Female students showed lower averages and less variability in these factors, suggesting a more consistent but modest self-assessment of their compassionate behavior. This may be influenced by societal expectations that encourage women to adopt caregiving roles and emphasize humility, potentially leading to more uniform expressions of empathy [7,8]. Male students, on the other hand, displayed greater variability and higher average scores, possibly reflecting a wider range of experiences and expressions of empathy, as well as confidence in self-assessment, consistent with studies indicating that men might express empathy in more varied forms due to less rigid societal expectations [6,8,25].

The findings also reveal that male students tended to have higher scores in GS, GRIP, and GRID. Higher scores in GS among male students could indicate a heightened awareness of gender issues, possibly due to specific training or personal interest in the subject, suggesting that targeted interventions can enhance gender sensitivity across genders [26]. The higher GRIP and GRID scores suggest that male students may hold more traditional views on gender roles, both for patients and doctors, potentially influenced by cultural and social norms [14,17].

These gender differences have significant implications for medical education. Training programs should be tailored to address the specific needs and characteristics of both male and female students. For example, encouraging male students to maintain their gender sensitivity while challenging traditional gender role ideologies can promote a more inclusive approach to patient care. For female students, fostering confidence in their compassionate care abilities and providing opportunities to express empathy in diverse ways can help overcome societal constraints [25,26].

Moving on to the analysis of the answers to the open-ended questions, we get an overview of perceptions about violence. Most responses indicate that physical, sexual, psychological, and verbal violence are considered the most common forms of violence. The responses reflect different opinions, but they tend to focus on characteristics such as aggression, manipulation, lack of empathy, and short-temperedness. These traits are often associated with the perception of a violent person. Concerning reactions to violence, responses suggest that many people would consider the option of reporting violence, seeking support from those close to them, and taking personal actions to protect themselves. Respondents share different opinions on the causes, but many identify factors such as ignorance, insecurity, past trauma, family problems, and social and cultural context as possible causes of violence. Concepts such as affective education, awareness-raising in educational institutions, and empathy emerge as protective factors against violence. These suggest that education and awareness can play a crucial role in preventing violence. Overall, the responses highlight the need to address violence in all its forms through education, awareness-raising, and empathy. Promoting concrete actions to denounce and prevent violence is therefore essential to creating a safer and more respectful society.

The findings of this study have substantial implications for the development of gender-sensitive medical education programs within university courses. Recognizing significant gender differences in empathy, gender role ideologies, and gender sensitivity among medical students can inform tailored approaches to teaching and curriculum design [27]. 

The nearly identical scores in perspective-taking between genders suggest that existing training methods effectively cultivate this aspect of empathy. However, the pronounced differences in compassionate care and walking in the patient’s shoes indicate a need for more targeted interventions. Female students’ modest self-assessment and uniform expressions of empathy highlight the importance of creating a learning environment that encourages diverse and confident expressions of empathy. Meanwhile, the greater variability and higher scores among male students suggest that addressing societal expectations and providing diverse opportunities for empathy expression could be beneficial.

Incorporating gender-sensitive training into medical curricula should emphasize the importance of recognizing and overcoming unconscious biases, ensuring that all patients receive equitable attention and care regardless of gender.

Additionally, the higher scores in GS among male students could be leveraged to foster peer-led discussions and mentoring programs where male students share their insights on gender issues. This peer interaction can create a more collaborative and supportive learning environment, encouraging all students to develop a deeper understanding of gender dynamics in healthcare.

To further enhance gender-sensitive medical education, qualitative methods such as interviews and focus groups should be incorporated to explore the lived experiences of medical students. Understanding the underlying factors that shape gendered perspectives and behaviors can provide valuable insights for curriculum development. 

Furthermore, longitudinal studies tracking changes in empathy, gender role ideologies, and gender sensitivity over time would offer a more comprehensive understanding of how these attributes evolve throughout medical training. This information can be used to continually refine and adapt training programs to better meet the needs of medical students.

By addressing these aspects of medical education, universities can cultivate a healthcare workforce that is not only empathetic and sensitive to gender issues but also equipped to provide personalized, inclusive, and fair medical care. This, in turn, will improve patient outcomes, foster trust in the doctor-patient relationship, and contribute to a more equitable healthcare system.

Suggestions for training aimed at medical students

Our comprehensive training program spans multiple phases, focusing on raising awareness of gender bias in medicine, enhancing empathetic skills for handling gender-based violence, and providing hands-on experience. Through workshops, research projects, simulations, and real-world observations, medical students are prepared to address gender-related issues with sensitivity and professionalism.

Phase 1: Training Activities on Gender Bias Awareness

Gender bias awareness workshop (duration: two hours): Workshop organization, where we conduct a focused workshop to increase awareness of gender bias in the medical field; Guest speakers, where we invite healthcare professionals and researchers to share their personal experiences and insights on gender bias; Case studies to analyze real-world examples highlighting gender bias in healthcare settings; Group discussions to encourage students to share their thoughts and ideas on addressing gender bias through facilitated group discussions; Resources to provide additional materials and recommended readings for further learning.

Research project on gender bias in medical education (duration: four weeks): Formation of research groups where students form groups to investigate gender bias in medical education; Literature review, where reviews are conducted of existing studies and articles relevant to gender bias; Survey design and distribution to create and distribute surveys or questionnaires to gather data from peers and faculty; Data analysis to examine the collected data to identify patterns and trends related to gender bias; Report preparation to compile findings and recommendations into a comprehensive report; Presentation of the research findings to the academic community, including faculty, administrators, and fellow students, to raise awareness and promote change.

Total duration of Phase 1: six hours

Phase 2: Training Activities on Doctors' Empathic Skills for Victims of Gender-Based Violence

Empathy as a core competency (duration: four hours): Empathetic interview simulation, which involves role-playing where students practice conducting empathetic interviews by role-playing as doctors and victims of gender-based violence and group reflection, where after the simulation, students discuss their experiences, emotions, and challenges, sharing strategies for providing empathetic support; Case study analysis, where students analyze various case studies involving victims of gender-based violence, considering the emotional and practical challenges faced by doctors; and group discussions, where students share their analyses and discuss empathetic approaches to support victims effectively.

Total duration of Phase 2: four hours

Phase 3: Training Activities on Gender-Based Violence

Comprehensive training program (duration: 10 weeks, two hours per week): The objective is to equip medical students with deep knowledge about gender-based violence, its health impacts, and the necessary skills to identify, manage, and support victims. Weekly meetings where each session integrates theoretical knowledge, case studies, group activities, and guided discussions.

Observation and practical experience: Anti-violence center observation, where students observe and interact with professionals at an anti-violence center, witnessing the handling of real cases and the provision of support; Emergency room observation, where students observe emergency room procedures for managing victims of gender-based violence, including assessments, injury documentation, and emergency management; Support group participation, where, under professional supervision, students participate in support groups, learning to understand and respond to victims' emotional needs; Case simulations where students engage in simulated cases of gender-based violence to practice communication, assessment, and victim management skills; Supervision and feedback where throughout the hands-on experiences, students receive supervision and feedback from experienced professionals, allowing them to reflect on their performance and improve their skills.

Total duration of Phase 3: 20 hours over 10 weeks

## Conclusions

The present study explored gender differences in empathy, gender role ideologies, and gender sensitivity among medical students, highlighting significant variations that can inform medical education and training programs. The results demonstrate no significant gender difference in perspective-taking (Factor 1), as male and female students scored nearly identically. This suggests that both genders possess a similar capacity for understanding others' perspectives, likely due to the general cognitive and emotional development emphasized in their education and socialization. This finding aligns with previous research indicating that perspective-taking is a fundamental component of empathy that may not be heavily influenced by gender-specific traits. Conversely, significant gender differences were observed in compassionate care (Factor 2) and walking in the patient’s shoes (Factor 3). Female students showed lower averages and less variability in these factors, suggesting a more consistent but modest self-assessment of their compassionate behavior. This may be influenced by societal expectations that encourage women to adopt caregiving roles and emphasize humility, potentially leading to more uniform expressions of empathy. Male students, on the other hand, displayed greater variability and higher average scores, possibly reflecting a wider range of experiences and expressions of empathy, as well as confidence in self-assessment.

The findings also reveal that male students tend to have higher scores in GS, GRIP, and GRID. Higher scores in GS among male students could indicate a heightened awareness of gender issues, possibly due to specific training or a personal interest in the subject. The higher GRIP and GRID scores suggest that male students may hold more traditional views on gender roles, both for patients and doctors, potentially influenced by cultural and social norms. These gender differences have significant implications for medical education. Training programs should be tailored to address the specific needs and characteristics of both male and female students. For example, encouraging male students to maintain their gender sensitivity while challenging traditional gender role ideologies can promote a more inclusive approach to patient care. For female students, fostering confidence in their compassionate care abilities and providing opportunities to express empathy in diverse ways can help overcome societal constraints.

The study's limitations include its focus on a specific geographic region and a single university, which may not represent broader contexts. Future research should examine these issues across various medical programs and geographical areas to enhance generalizability. Additionally, the cross-sectional design precludes an assessment of how these attributes evolve throughout medical training and professional practice. Longitudinal studies are needed to track changes over time and provide deeper insights.

Future studies should also incorporate qualitative methods, such as interviews and focus groups, to explore the underlying factors and lived experiences shaping gendered perspectives and behaviors among medical students. Investigating the impact of specific pedagogical approaches and training interventions on these outcomes is crucial. Ultimately, addressing these limitations and conducting more comprehensive investigations will enable the development of more effective strategies to cultivate a healthcare workforce that is truly gender-sensitive and equipped to provide equitable, empathetic care to all patients. By enhancing our understanding of gender differences in empathy and sensitivity, medical educators can better prepare future physicians to address gender-based violence and other gender-related health issues, ensuring personalized, inclusive, and fair medical care.
